# Safety analysis of omitting axillary lymph node dissection in early-stage breast cancer with 1–2 sentinel lymph nodes macro-metastases: a meta-analysis

**DOI:** 10.3389/fonc.2025.1620034

**Published:** 2025-09-25

**Authors:** Yu Chen, Xiaoming Zhang, Qingping Wu, Shanshan Gao, Lu Wang, Minxia Zeng, Lihu Gu, Changrui Sheng

**Affiliations:** ^1^ The Graduate School, Zhejiang Chinese Medical University, Hangzhou, Zhejiang, China; ^2^ The First Clinical Medical College, Zhejiang Chinese Medical University, Hangzhou, Zhejiang, China; ^3^ Department of Ultrasound, Ningbo No. 2 Hospital, Ningbo, Zhejiang, China; ^4^ Department of General Surgery, Ningbo No. 2 Hospital, Ningbo, Zhejiang, China

**Keywords:** omitting, early-stage breast cancer, sentinel lymph node biopsy, axillary lymph node dissection, macro-metastases, meta-analysis

## Abstract

**Background:**

Currently, the axillary management strategy of omitting axillary lymph node dissection (ALND) in early-stage breast cancer (BC) patients with cT1-2, clinically node-negative (cN0), and sentinel lymph node biopsy (SLNB) revealing 1–2 sentinel lymph nodes (SLNs) macro-metastases remains controversial. This study aims to systematically evaluate the safety of omitting ALND in this population.

**Methods:**

This study was conducted in accordance with the Preferred Reporting Items for Systematic Reviews and Meta-Analyses (PRISMA) guidelines and was registered with the registration number: CRD42025645388. A systematic literature search was conducted across five electronic databases (PubMed, Web of Science, Cochrane Library, Ovid Medline, and Embase) from inception through December 2024. Randomized controlled trials (RCTs) and cohort studies meeting the predefined eligibility criteria were included. Primary outcomes included disease-free survival (DFS) and overall survival (OS). The association between ALND omission and long-term outcomes was assessed using pooled hazard ratios (HRs) with 95% confidence intervals (CIs).

**Results:**

Fifteen studies (6 RCTs, 9 cohort studies) involving 33,599 patients in the SLNB-only group and 95,711 controls receiving SLNB+ALND were analyzed. No significant differences in DFS (HR = 0.99, 95%CI:0.85-1.14, p=0.857) or OS (HR = 1.03, 95%CI: 0.92-1.14 p=0.251) were observed in both groups. Subgroup analyses by follow-up duration (5-years and 10-years), study design (RCTs and cohort studies), and region (Eastern and Western) showed no survival differences between the experimental and control groups. (all p values are greater than 0.05).

**Conclusion:**

Omitting ALND is safe for early-stage BC patients with cT1-2, cN0, and 1–2 SLNs macro-metastases.

**Systematic review registration:**

https://www.crd.york.ac.uk/prospero/, identifier CRD42025645388.

## Introduction

Breast cancer (BC) is the second most common cancer globally and the most frequently diagnosed cancer in women, accounting for 25% of all female cancer cases. It ranks fourth among cancer-related deaths worldwide and is responsible for 16.7% of female cancer-related fatalities (1). Current therapeutic modalities for BC encompass radiation therapy, chemotherapy, targeted therapy, immunotherapy, and endocrine therapy, while surgical intervention remains the cornerstone of curative treatment. Within surgical oncology, axillary lymph node dissection (ALND) has historically held significant prognostic importance. It is well known that axillary lymph nodes are an important pathway for BC metastasis, making precise axillary nodal staging essential for both prognostic evaluation and therapeutic decision-making (2). ALND can remove potentially metastatic lymph nodes, provides definitive axillary staging and improve long-term prognosis, and it has long been the standard surgical procedure for BC (3). However, ALND carries substantial postoperative morbidity including lymphedema, sensory nerve injury, infection, and hemorrhage, all of which adversely impact patients’ quality of life (4). Early-stage BC is typically defined as primary tumors classified as T1-2, regional lymph node status N0-N1, and without distant metastasis (5). With the widespread application of screening technologies, the detection rate of early-stage BC has significantly increased, leading to markedly improved patient survival outcomes (6). A major clinical challenge in contemporary BC management is to achieve accurate axillary staging while minimizing surgical trauma and optimizing quality of life - specifically, by reducing unnecessary ALND.

The advent of sentinel lymph node biopsy (SLNB) in the 1990s revolutionized axillary staging, mitigating these morbidity concerns while maintaining oncological safety (7). Relevant studies demonstrated a 97.5% concordance rate between sentinel lymph nodes (SLNs) status and axillary lymph node pathology, with SLNB achieving diagnostic accuracy rates up to 97% (8). These findings support the omission of ALND in patients with negative SLNB results (9). To date, ALND remains standard care for positive SLNB cases. However, in relevant studies, it was found that among early-stage BC patients cT1-2, clinically node-negative (cN0) and SLNB positive who underwent ALND, approximately 75.7% had only one positive SLN (10). In other words, for such patients, the clinical value of ALND is somewhat limited. Subsequent randomized controlled trials (RCTs) including Z0011, SENOMAC, and AMAROS have rigorously investigated this clinical dilemma (11–13). The results showed that for early-stage BC patients who were cT1-2, cN0 and had 1–2 positive SLNs after SLNB, omitting ALND was not inferior to ALND in terms of prognosis. While existing meta-analyses have explored the safety of omitting ALND in early-stage BC patients, limitations persist. Most studies focus on populations with micro-metastases or isolated tumor cells (ITC) (for this population, the axillary node disease burden is low, indicating a favorable prognosis), constrained by insufficient sample sizes and further subgroup stratification (14, 15). Therefore, this study, through a systematic review of published RCTs and cohort studies, aims to explore the impact of omitting ALND on long-term prognosis in early-stage BC patients who are cT1-2, cN0 and 1–2 SLNs macro-metastatic after SLNB.

## Methods

### Search strategy

This meta-analysis, following the Preferred Reporting Items for Systematic Reviews and Meta-Analyses (PRISMA) guidelines (16), was registered in the international Prospective Register of Systematic Reviews (PROSPERO) under the number CRD42025645388. A literature search was conducted across five electronic databases: PubMed, Web of Science, Cochrane Library, Ovid Medline, and Embase. The search spanned from the inception of each database to December 2024. The search strategy combined Medical Subject Headings (MeSH) terms and free-text terms related to “breast neoplasms” and “sentinel lymph node”. The search string was as follows: ((((((((((((((((breast neoplasms [mesh]) OR (breast tumor)) OR (breast cancer)) OR (cancer of breast)) OR (malignant neoplasm of breast)) OR (malignant tumor of breast)) OR (mammary cancer)) OR (mammary neoplasms, human)) OR (breast carcinoma)) OR (human mammary carcinoma)) OR (breast carcinoma *in situ*)) OR (carcinoma, ductal, breast)) OR (carcinoma, lobular)) OR (inflammatory breast neoplasms)) OR (triple negative breast neoplasms)) OR (inflammatory breast neoplasms)) AND ((sentinel lymph node [mesh]) OR (sentinel lymph nodes)) OR (sentinel node))).

The search terms and strategies were adjusted according to the indexing systems and requirements of each database. To avoid overlooking potential studies, a secondary manual search was conducted on the references of the included articles.

### Inclusion criteria and exclusion criteria

Articles were considered for inclusion only if they fulfilled the following criteria defined by the PICOS principle: (1) patients were diagnosed with early-stage BC, with clinical staging of cT1-2, cN0, and M0; (2) all patients underwent SLNB, with pathological results demonstrating macro-metastases in 1–2 positive SLNs; (3) the experimental group underwent SLNB alone without subsequent ALND; (4) the control group underwent SLNB followed by ALND; (5) studies reported long-term outcomes, including overall survival (OS) and disease-free survival (DFS); (6) study design included RCTs and cohort studies.

The exclusion criteria were as follows: (1) data were unavailable; (2) relevant outcomes of interest were not reported; (3) full-text articles could not be obtained; (4) articles were not in English; (5) when articles with data updates were available, only the most recent and/or the most comprehensive ones were considered for inclusion.

### Data extraction and quality assessment

Two independent investigators extracted data using standardized forms capturing: first author, publication year, study design, enrollment period, surgical procedures, follow-up duration, sample characteristics (SLN status, molecular subtypes, histologic grades), and survival outcomes.

For RCTs, methodological quality was evaluated using the Cochrane Risk of Bias Tool 1.0 (RoB 1.0) (17). This instrument assesses potential biases across multiple domains, including randomization sequence generation, allocation concealment, blinding of participants/investigators, blinding of outcome assessment, completeness of outcome data, and selective reporting. Risk of bias was categorized as “high”, “low”, or “unclear”. For cohort studies, methodological rigor was assessed via the Newcastle-Ottawa Scale (NOS), which evaluates three domains: selection bias, comparability, and outcome measurement. A scoring system was applied to each domain, with studies achieving NOS scores≥6 classified as high-quality. Discrepancies between reviewers were resolved through consensus discussion during the data extraction and quality appraisal processes.

### Statistical analysis

Meta-analyses were conducted using Stata 12.0 and Review Manager 5.3. The association between omission of ALND and long-term prognosis was evaluated by calculating pooled hazard ratios (HRs) with 95% confidence intervals (CIs). Effect estimates were interpreted as favoring the experimental group when HR<1 and the control group when HR>1. Heterogeneity was assessed using Cochran’s Q statistic (χ² test) and I² quantification, with I² values>50% indicating substantial heterogeneity and ≤ 50% indicating low heterogeneity (18). Subgroup analyses stratified by follow-up times, study design, and geographic region were performed to investigate potential sources of heterogeneity.

Given anticipated heterogeneity due to variations in population characteristics, ethnicities, treatment protocols, and BC subtypes, a random-effects model was uniformly applied to enhance result reliability. Sensitivity analyses were conducted by sequentially excluding each study to evaluate result stability. Potential publication bias was examined using Begg’s test (19). All analyses employed two-tailed tests, with statistical significance defined at p<0.05.

## Results

### Study selection

**Figure 1:**
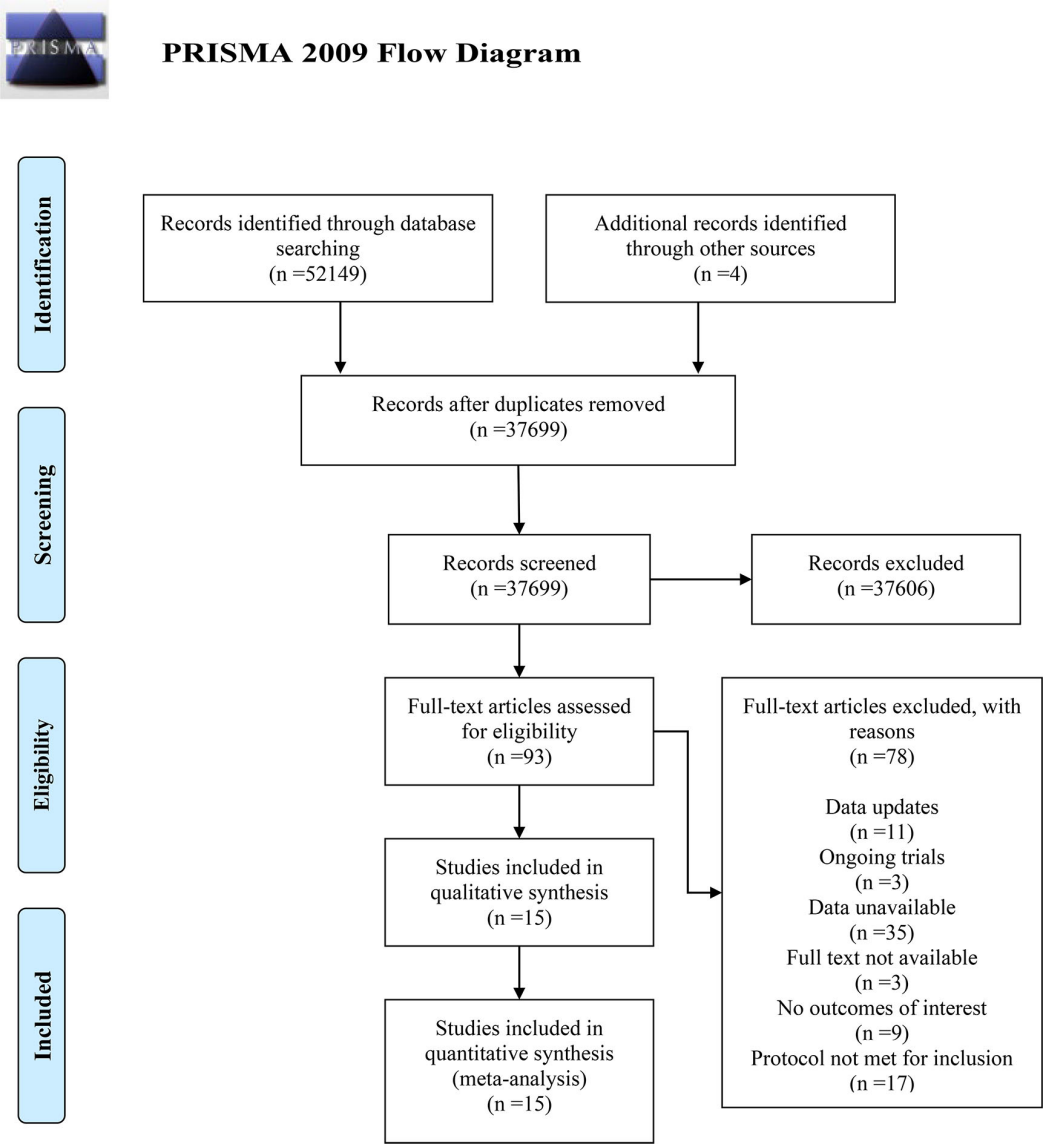
Flow diagram containing details of study selection.

A total of 52,153 terms were identified through the initial search of five electronic databases and additional secondary manual indexing. After duplicates were removed using Note Express 4.0 software, 37,699 terms remained. By reading the titles and abstracts, 37,606 terms were excluded. The full texts of the remaining 93 articles were reviewed, and 78 were excluded for the following reasons: updated data (n=11); unavailable data (n=35); full text unobtainable (n=3); lack of interesting outcomes (n=9); methodological ineligibility (n=17); and ongoing studies (n=3). Ultimately, 15 articles were included in the meta-analysis. The detailed process of inclusion and exclusion is shown in the PRISMA flow diagram (**Figure 1**).

### Study characteristics

The 15 included articles, comprising 6 RCTs (11–13, 20–22)and 9 cohort studies (23–31), published between 2009 and 2024, were mostly conducted in Western countries. The recruitment period ranged from 1998 to 2021, with more than 33,000 participants in the experimental group and more than 95,000 in the control group. The recruited patients generally suffered from early-stage BC and were characterized by cT1-2, cN0, M0, and had 1–2 positive SLNs after SLNB. Surgical procedures included breast-conserving surgery and mastectomy. Specific information is presented in **Table 1**. Further details regarding the median follow-up time of the included studies, the median age of patients at recruitment, the distribution of the number of positive SLNs, and the distribution of histological grading are summarized in **Supplementary Tables 1–1**, **1-2**.

**Table 1:** Characteristics of the studies included in this meta-analysis.

Author	Year	Country	Recruiting time	Experiment arm	Control arm	Primary outcomes	Inclusion criteria	Study design
Bartels SAL (13)	2023	Multicenter	2001-2010	681	744	OS DFS	cN0, cT1-2, positive SLNs	RCTs
De Boniface J (12)	2024	Multicenter	2015-2021	1335	1205	DFS	cN0, cT1-3, 1–2 positive SLNs	RCTs
Tinterri C (20)	2022	Italy	2015-2020	440	439	OS DFS	cN0, cT1-2, 1–2 positive SLNs, 40–75 years	RCTs
Giuliano AE (11)	2017	Multicenter	1999-2004	446	445	OS DFS	cN0, cT1-2, 1–2 positive SLNs, BCS	RCTs
Canavese G (21)	2016	Italy	1998-2001	110	115	OS DFS	cN0, cT1-2, 18–75 years	RCTs
Sávolt Á (22)	2017	Hungary	2002-2009	230	244	OS DFS	cN0, cT1-3, positive SLNs	RCTs
Zhao X (23)	2024	China	2016-2021	234	234	OS DFS	cN0, cT1-2, 1–2 positive SLNs, Women, ≥18years, mastectomy	Cohort studies
Schwieger L (24)	2024	American	2010-2017	8427	11574	OS	cN0, cT1-2, 1–3 positive SLNs, women, mastectomy	Cohort studies
de Wild SR (25)	2024	Netherlands	2013-2014	219	437	DFS	cN0, cT1-2, ≤3 positive SLNs, women, ≥18years, mastectomy	Cohort studies
Joo JH (26)	2019	Korea	2000-2015	158	1539	OS DFS	cN0, 1–3 positive SLNs, women, mastectomy	Cohort studies
Sanvido VM (27)	2021	Brazil	2008-2018	56	41	OS	cN0, T1-2, 1–3 positive SLNs, BCS	Cohort studies
Sun J (28)	2021	American	1999-2018	128	201	OS DFS	cN0,1–3 positive SLNs	Cohort studies
Jung J (29)	2019	Korea	2010-2016	707	990	DFS	cN0, cT1-2, 1–2 positive SLNs, women, BCS	Cohort studies
Arisio R (30)	2019	Italy	2004-2014	211	406	DFS	cN0, positive SLNs,	Cohort studies
Bilimoria KY (31)	2009	American	1998-2000	20217	77097	OS DFS	cN0, cT1-3, positive SLNs, ≥18 years	Cohort studies

OS, overall survival; DFS, disease-free survival; BCS, breast conserving surgery; SLNs, sentinel lymph nodes, RCTs, randomized controlled trials.

### Methodological quality of included studies

The RoB 1.0 was used to evaluate the quality of RCTs, revealing a high risk of bias due to the nature of the interventions (mainly because blinding was not performed). The risk of bias assessment for RCTs is detailed in **Supplementary Figures 1**, **2**. The NOS as used to evaluate cohort studies, with all studies rated as moderate to high quality, scoring at least 7 out of 9 points. Specific scores are shown in **Supplementary Table 2**.

### Long-term prognosis

#### DFS

**Figure 2:**
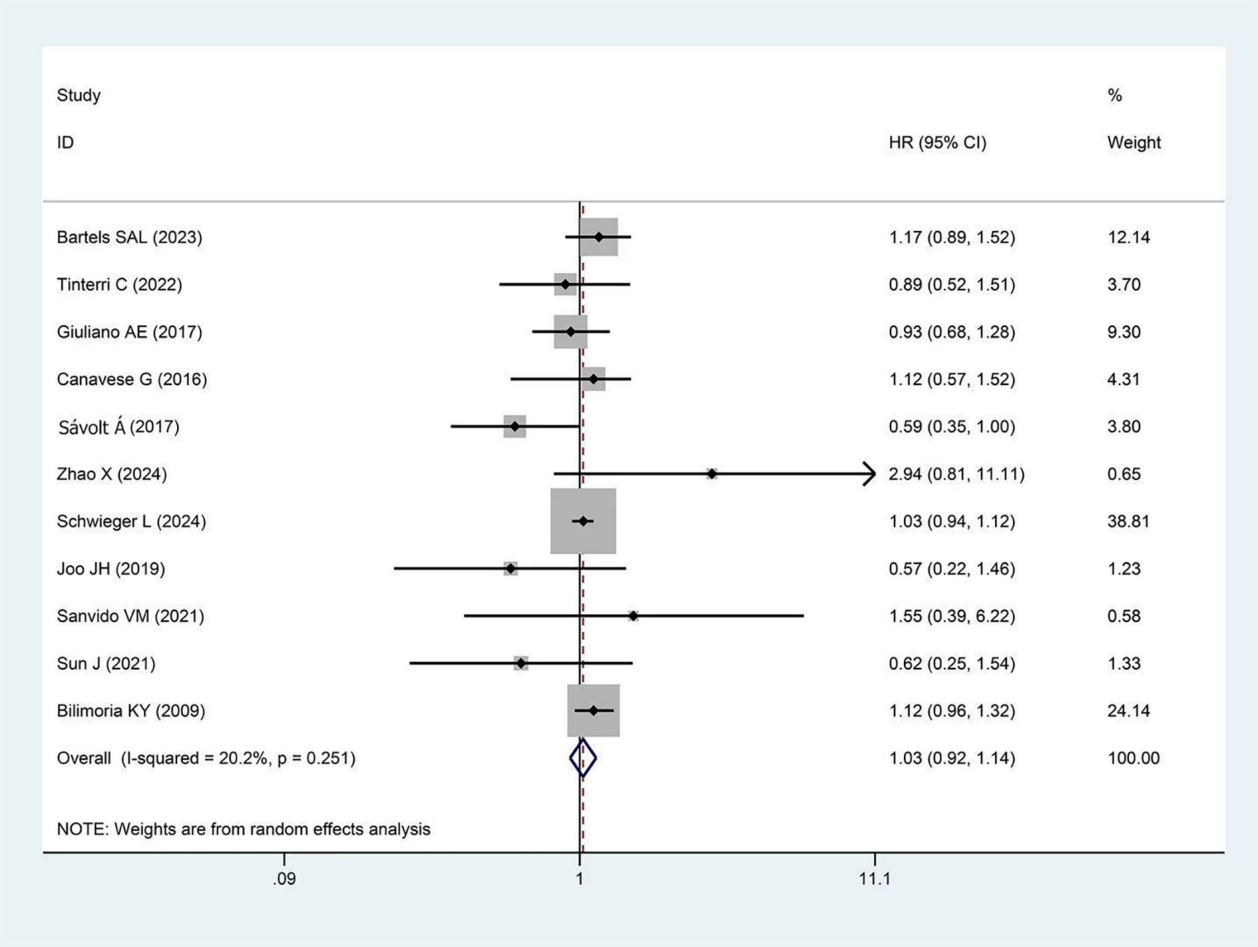
Forest plot showing the effect of omitting ALND on DFS in early-stage BC cT1-2, cN0, and 1–2 SLNs macro-metastases (p=0.857).

A total of 13 studies reported DFS, with more than 25,000 participants in the experimental group and more than 84,000 in the control group. The pooled results showed no significant difference in DFS between the two groups (HR = 0.99, 95%CI:0.85-1.14, p=0.857, I²=20.5%) as presented in **Figure 2**.

Subgroup analysis results are shown in **Table 2**. Studies were divided into 5-year and 10-year subgroups based on follow-up duration. No statistical difference in DFS was observed in either the 5-year subgroup (HR = 1.02, p=0.827) or the 10-year subgroup (HR = 0.95, p=0.694) between the experimental and control groups. When stratified by study design, no significant difference in DFS was observed between the experimental and control groups in the RCTs subgroup (HR = 1.01, p=0.934) or the cohort studies subgroup (HR = 1.02, p=0.906). In terms of geographical region, studies were divided into Eastern and Western subgroups. Neither the Eastern subgroup (HR = 0.96, p=0.866) nor the Western subgroup (HR = 1.00, p=0.957) showed a statistical difference in DFS between the experimental and control groups. Sensitivity analysis demonstrated the stability of the pooled DFS results (**Supplementary Figure 3**). Moreover, Begg’s test indicated no significant publication bias (p=0.951, **Supplementary Figure 4**).

**Table 2:** Subgroup analysis of disease‐free survival.

Subgroup	No. of studies	HR	95%CI	P	Heterogeneity	
					I^2^	P
Duration of follow-up						
5 years	11	1.02	0.88-1.18	0.827	11.9%	0.331
10 years	4	0.95	0.74-1.23	0.694	48.8%	0.119
Study design						
RCTs	6	1.01	0.88-1.15	0.934	0%	0.426
Cohort studies	7	1.02	0.74-1.40	0.906	41.0%	0.118
Region						
Eastern	3	0.96	0.62-1.50	0.866	48.0%	0.146
Western	9	1.00	0.85-1.17	0.957	25.5%	0.217

HR, hazard ratio; CI, confidence interval; RCTs, randomized controlled trials.

#### OS

**Figure 3:**
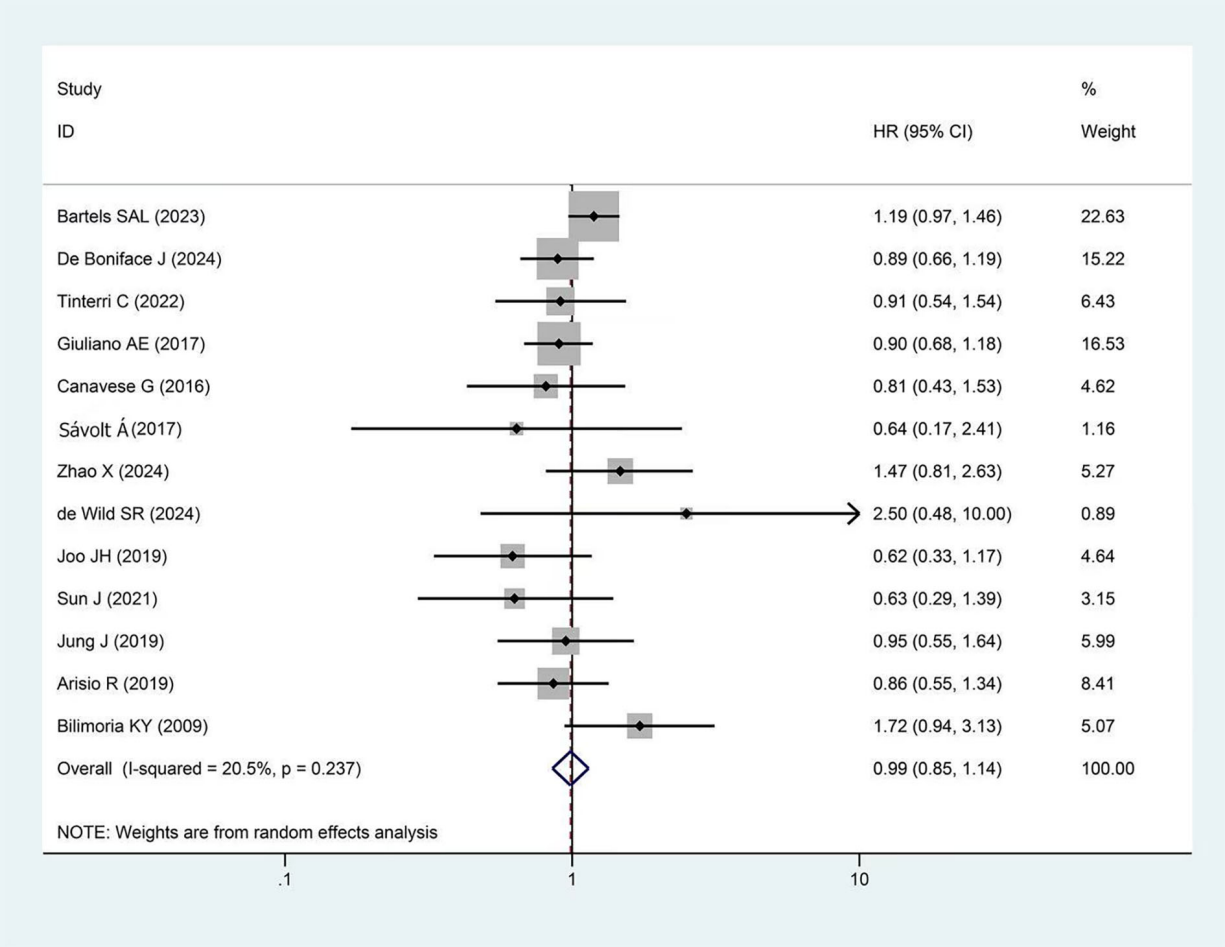
Forest plot showing the effect of omitting ALND on OS in early-stage BC cT1-2, cN0, and 1–2 SLNs macro-metastases (p=0.606).

A total of 11 studies reported OS, with more than 31,000 participants in the experimental group and more than 92,000 in the control group. The pooled results showed no significant difference in OS between the two groups (HR = 1.03, 95%CI: 0.92-1.14 p=0.251, I²=20.2%) as presented in **Figure 3**.

Subgroup analysis results are shown in **Table 3**. Based on follow-up duration, studies were divided into 5-year (HR = 1.02, p=0.776) and 10-year subgroups (HR = 1.05, p=0.635). In terms of study design, studies were divided into RCTs (HR = 0.96, p=0.735) and cohort study subgroups (HR = 1.05, p=0.425). Studies were also divided into Western (HR = 1.05, p=0.202) and Eastern subgroups (HR = 1.21, p=0.813). In the subgroup analyses mentioned above, no significant statistical differences were observed in OS between the experimental and control groups. Sensitivity analysis demonstrated the stability of the pooled OS results. (**Supplementary Figure 5**). Additionally, Begg’s test showed no significant publication bias (p=0.640, **Supplementary Figure 6**).

**Table 3:** Subgroup analysis of overall survival.

Subgroup	No. of studies	HR	95%CI	P	Heterogeneity	
					I^2^	P
Duration of follow-up						
5 years	9	1.02	0.90-1.15	0.776	28.6%	0.191
10 years	4	1.05	0.87-1.26	0.635	0%	0.414
Study design						
RCTs	5	0.96	0.78-1.19	0.735	30.8%	0.216
Cohort studies	6	1.05	0.93-1.20	0.425	21.7%	0.271
Region						
Eastern	2	1.21	0.24-6.03	0.813	74.8%	0.046
Western	7	1.05	0.98-1.12	0.202	0%	0.713

HR, hazard ratio; CI, confidence interval; RCTs, randomized controlled trials.

## Discussion

In early-stage BC, lymphatic dissemination represents the predominant metastatic pathway, with approximately 33% of patients presenting with regional lymph node involvement at initial diagnosis (32). The axillary lymph nodes, due to their anatomical location and lymphatic drainage characteristics, are the primary target organs for metastasis. Traditional ALND was once the standard procedure for blocking metastasis and assessing axillary status. However, its complications significantly affect patients’ postoperative quality of life. With the development of SLNB technology, characterized by ≥98% accuracy in axillary staging and a lower risk of complications, it has become the preferred method for axillary staging (33). For patients with negative SLNs, SLNB has become the standard treatment when combined with comprehensive systemic therapy. For patients with positive SLNs, ALND is still required.

Lymph node metastasis can be classified into three types based on the diameter of tumor cells in the lymph nodes: ITC (≤0.2 mm), micro-metastasis (>0.2 mm and ≤2 mm), and macro-metastasis (>2 mm) (34). Currently, patients with positive SLNs routinely undergo ALND, but recent studies have revealed potential for optimizing this strategy. In cN0 patients and positive SLNs after SLNB, data show that in about 60% of cases, only positive SLNs is found during ALND, with no other lymph node metastasis (35). Further research by Sonia Martinez Alcaide’s team found that among patients with macro-metastasis in SLNs, 66.7% had only SLN involvement after ALND; 7.9% had one non-sentinel lymph node (NSLN) involved; 6.3% had two NSLNs involved; and 19% had three or more involved NSLNs (36). In patients with micro-metastasis, a lower axillary metastatic burden was observed: 83.9% had only SLN involvement, 12.9% had one NSLN involved, and 3.2% had two NSLNs involved. This finding was confirmed by the long-term follow-up results of the IBCSG 23–01 study (37). Specifically, in early-stage BC patients who were cT1-2, cN0, and had micro-metastasis (including ITC) in SLNB, there were no statistically significant differences in OS, DFS, or regional control between patients who received ALND and those who underwent SLNB alone. The latest European Society for Medical Oncology (ESMO) guidelines (38) and multiple meta-analyses (14, 39) also suggest that for patients with the least axillary metastatic burden, SLNB alone is sufficient.

The number of pathologically SLNs in ALND demonstrated a significant positive correlation with NSLN positivity rates (40, 41). Among patients with 1–3 positive SLNs, the NSLN positivity rate was 37.9%; however, this rate increased dramatically to 83.3% when ≥4 SLNs were involved. It is worth noting, even in patients with 1–2 macro-metastases in SLNs, the overall axillary metastatic burden remains low, prompting exploration of whether ALND can be safely omitted for such patients. The 5-year follow-up results of the SENOMAC trial demonstrated that omitting ALND in patients with macro-metastases was non-inferior to ALND in terms of survival outcomes (12), a finding that aligns with the conclusions of this meta-analysis. No significant differences in DFS and OS were observed between the patients underwent ALND and those receiving SLNB alone. This may be due to the following reasons: 1. specific patients: as previously mentioned, the axillary burden in BC patients is low, with 60% of patients having tumor cells involving only SLNs (35), which is removed through SLNB. 2. Postoperative radiotherapy: most patients received postoperative radiotherapy, which effectively clears potential occult lesions in breast tissue, chest wall, or regional lymph nodes, reducing the risk of local recurrence (42). Therefore, even if there are residual positive lymph nodes after SLNB, postoperative regional lymph node radiotherapy can provide effective salvage. By the way studies have also indicated that for women with 1–3 positive lymph nodes, regional lymph node radiotherapy reduces the absolute risk of 15-year BC mortality by approximately 2-3% (43). 3. Postoperative adjuvant systemic therapy: at least 90% of patients received adjuvant systemic therapy, including chemotherapy, as well as targeted, immunotherapy, and endocrine therapy based on the molecular subtypes of BC. Current evidence suggests that BC is a disease with systemic tendencies, and even in early-stage BC, there may be microscopic metastases (44, 45). Adjuvant systemic therapy is crucial for improving patient prognosis. Studies have shown a 50% reduction in the 10-year recurrence rate and a 20% decrease in the 20-year risk of breast cancer mortality, significantly improving survival rates (46). Additionally, personalized treatment strategies are available based on different BC subtypes. For human epidermal growth factor receptor 2-positive (HER2-positive) BC patients, targeted therapy is available. Relevant studies have shown that compared to the observation group, patients receiving one year of trastuzumab treatment had a 12-year OS rate increased from 73% to 79% (47). For hormone receptor-positive (HormR-positive) BC, endocrine therapy can be used. Tamoxifen can reduce the risk of recurrence by about 50% and the risk of death by about 28% in estrogen receptor-positive (ER-positive) patients (48). For the most aggressive triple-negative breast cancer (TNBC), immunotherapy can be used. For instance, pembrolizumab combined with chemotherapy as adjuvant therapy has significantly improved OS. In the KEYNOTE-522 trial, with a median follow-up of 75.1 months, the 5-year OS rate was 86.6% in the pembrolizumab-chemotherapy group compared to 81.7% in the placebo-chemotherapy group, and the 5-year event-free survival rates were 81.2% and 72.2%, respectively (49). Postoperative adjuvant systemic therapy effectively targets systemic microscopic metastases, reduces recurrence risk, and improves survival rates, providing personalized treatment strategies for patients with different molecular subtypes and significantly enhancing BC survival rates.

BC subtypes demonstrate distinct recurrence timelines (32, 50). Luminal A subtype BC generally carries a low risk of recurrence, but some patients may experience delayed metastasis over 10 years post-diagnosis. Luminal B subtype BC typically exhibits a higher risk of recurrence within the first 5 years following diagnosis. HER2-positive BC often presents with the highest risk of recurrence within the first 3–5 years post-diagnosis. TNBC demonstrates the most aggressive early recurrence pattern, usually peaking within the first 3 years after diagnosis. Additionally, research indicates that the risk of BC recurrence can persist for 10 to 32 years (51). Therefore, for strategies that omitting ALND, long-term recurrence risk assessment and management must be considered. This study divided the included articles into 5-year and 10-year subgroups. Fortunately, no significant differences were observed in OS and DFS between the experimental group and the control group in either the 5-year subgroup or the 10-year subgroup. Studies were categorized into RCTs and cohort studies subgroups, with no statistically significant differences in clinical outcomes observed between these two groups. This study also examined the differences between Eastern and Western patients, including age at onset, subtype distribution, and genetic factors (52, 53). In Western countries, BC typically occurs at a later age, predominantly after 60 years old, whereas in Eastern regions, the peak incidence age is approximately 50 years old. Regarding subtype distribution: the most prevalent molecular subtype globally is HormR-positive/HER2-negative. However, in Eastern regions, the incidence of TNBC and HER2-positive BC is relatively higher. Concerning genetic factors: family history accounts for a larger proportion of BC cases in Western countries, with certain hereditary syndromes such as breast cancer susceptibility gene (BRCA) related breast and ovarian cancer being more common. In contrast, while family history remains an important risk factor in Eastern regions, its contribution to overall BC cases is relatively lower, and the types and characteristics of genetic syndromes differ from those observed in Western populations. Considering these differences, this study divided the included articles into Eastern and Western subgroups, and the results showed no significant differences. All results indicate that for early-stage BC, cT1-2, cN0, and with 1–2 macro-metastases after SLNB, omitting ALND may be safe.

The advantages of this study are: (1) this is the most comprehensive systematic meta-analysis to date for SLNs macro-metastases, proving the safety of omitting ALND. (2) Subgroup analyses were set based on follow-up time, study type, and region. (3) High-quality RCTs and well-designed cohort studies were included, with high credibility and a large sample size.

The disadvantages are: (1) subgroup analyses based on molecular subtypes are missing, and the potential impact of different molecular subtypes on axillary treatment strategies has not been clarified. (2) The maximum follow-up duration in this study was 10 years; however, certain subtypes (e.g., Luminal A) may require extended follow-up beyond this period. The absence of data beyond 10 years limits the assessment of long-term safety, thereby restricting the generalizability and clinical applicability of the findings. (3) The number of included Eastern studies is limited, and the study conclusions cannot yet be directly applied globally. (4) Heterogeneity in clinical practice patterns across different regions and time periods-particularly in surgical techniques and adjuvant treatment protocols may influence patient prognosis, thereby restricting the generalizability of our findings to a global population. (5) The lack of blinding in the included RCTs and regional differences in imaging and pathology standards for assessing recurrence may introduce subjectivity into DFS evaluation, potentially biasing the results. The findings of this study still require validation through larger sample sizes and multicenter randomized controlled trials.

This study shows that omitting ALND is safe for specific BC patients. In the future, axillary surgery for BC may be further downscaled. Emerging large-scale RCTs like INSEMA (54) and SOUND (55) have initially confirmed the feasibility of omitting SLNB in certain patients. These advancements promise to enhance post-operative quality of life for BC patients.

## Conclusion

This study demonstrates that for early-stage BC, cT1-2, cN0, and 1–2 SLN macro-metastases, omitting ALND is safe. This conclusion remains robust across varying follow-up periods, differing evidence hierarchies, and diverse geographic regions.

## Data Availability

The original contributions presented in the study are included in the article/**Supplementary Material**. Further inquiries can be directed to the corresponding authors.

## Supplementary material

Supplementary Figure 1 Risk of bias assessment of included RCTs studies.

Supplementary Figure 2 Summary of risk of bias across RCTs studies.

Supplementary Figure 3 Sensitivity analysis of DFS.

Supplementary Figure 4 Evaluation of publication bias in DFS studies(p=0.951).

Supplementary Figure 5 Sensitivity analysis of OS.

Supplementary Figure 6 Evaluation of publication bias in OS studies(p=0.640).

## Glossary

ALND: axillary lymph node dissection
BC: breast cancer
BRCA: breast cancer susceptibility gene
CI: confidence interval
cN0: clinically node-negative
DFS: disease-free survival
ER: estrogen receptor
ESMO: European Society for Medical Oncology
HER2: human epidermal growth factor receptor 2
HR: hazard ratio
ITC: isolated tumor cells
HormR: hormone receptor
MeSH: Medical Subject Headings
NOS: Newcastle-Ottawa Scale
NSLN: non-sentinel lymph node
OS: overall survival
PRISMA: Preferred Reporting Items for Systematic Reviews and Meta-Analyses
PROSPERO: International Prospective Register of Systematic Reviews
RCT: randomized controlled trial
RoB1.0: Cochrane risk of bias tool 1.0
SLN: sentinel lymph node
SLNB: sentinel lymph node biopsy
TNBC: triple-negative breast cancer
