# Supplementary figures and images for: Safety analysis of omitting axillary lymph node dissection in early-stage breast cancer with 1–2 sentinel lymph nodes macro-metastases: a meta-analysis
Source: Front Oncol. 2025 Sep 25;15:1620034. doi: 10.3389/fonc.2025.1620034 (PMC12507570; doi:10.3389/fonc.2025.1620034)

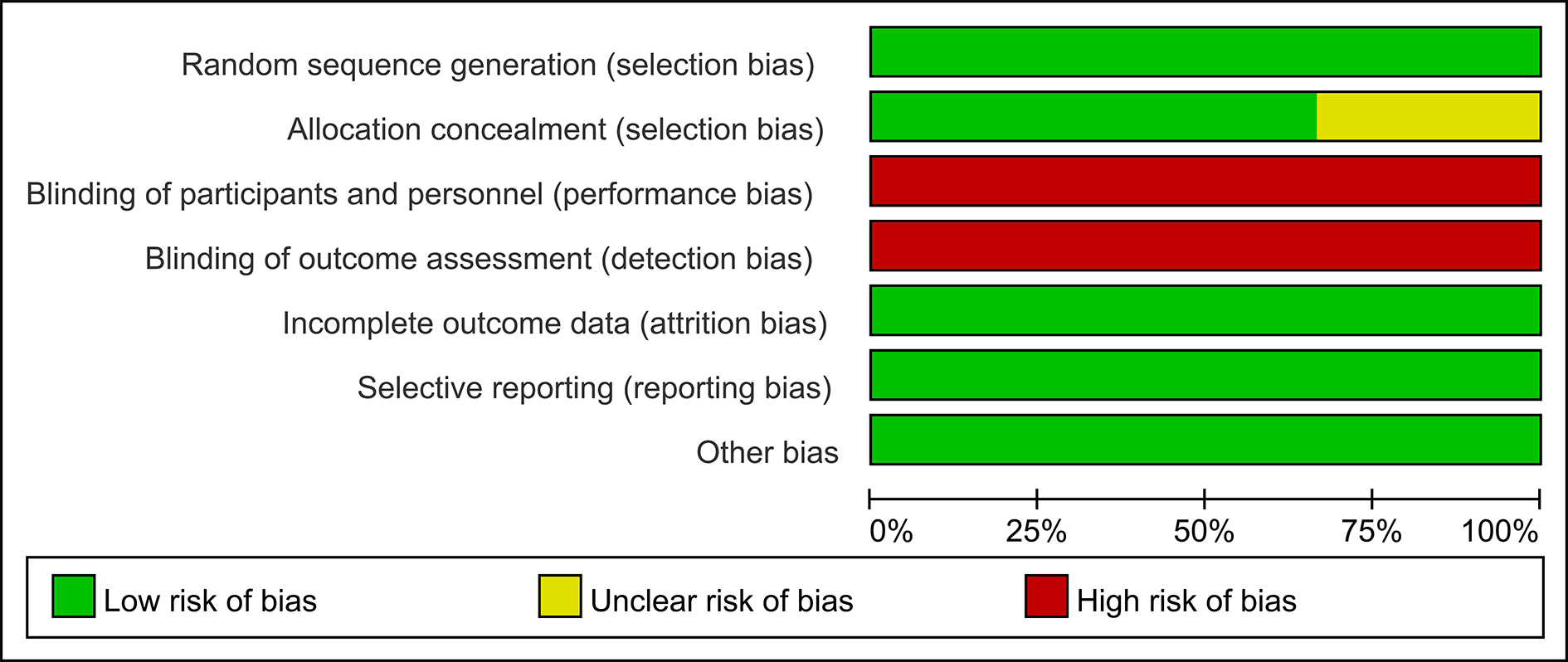

Supplement: Supplementary Figure 1 — Risk of bias assessment of included RCTs studies. [file Image1.tif]

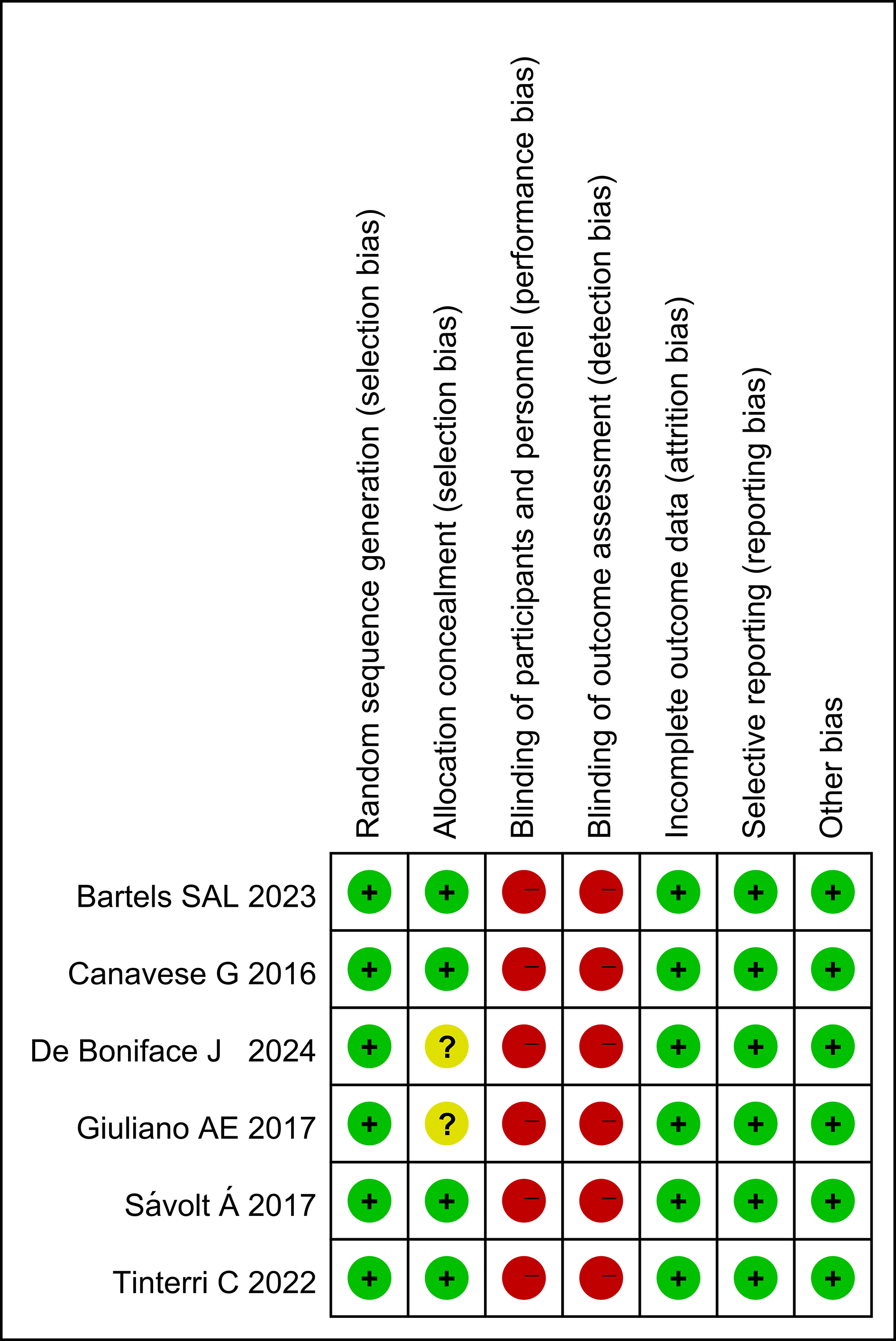

Supplement: Supplementary Figure 2 — Summary of risk of bias across RCTs studies. [file Image2.tif]

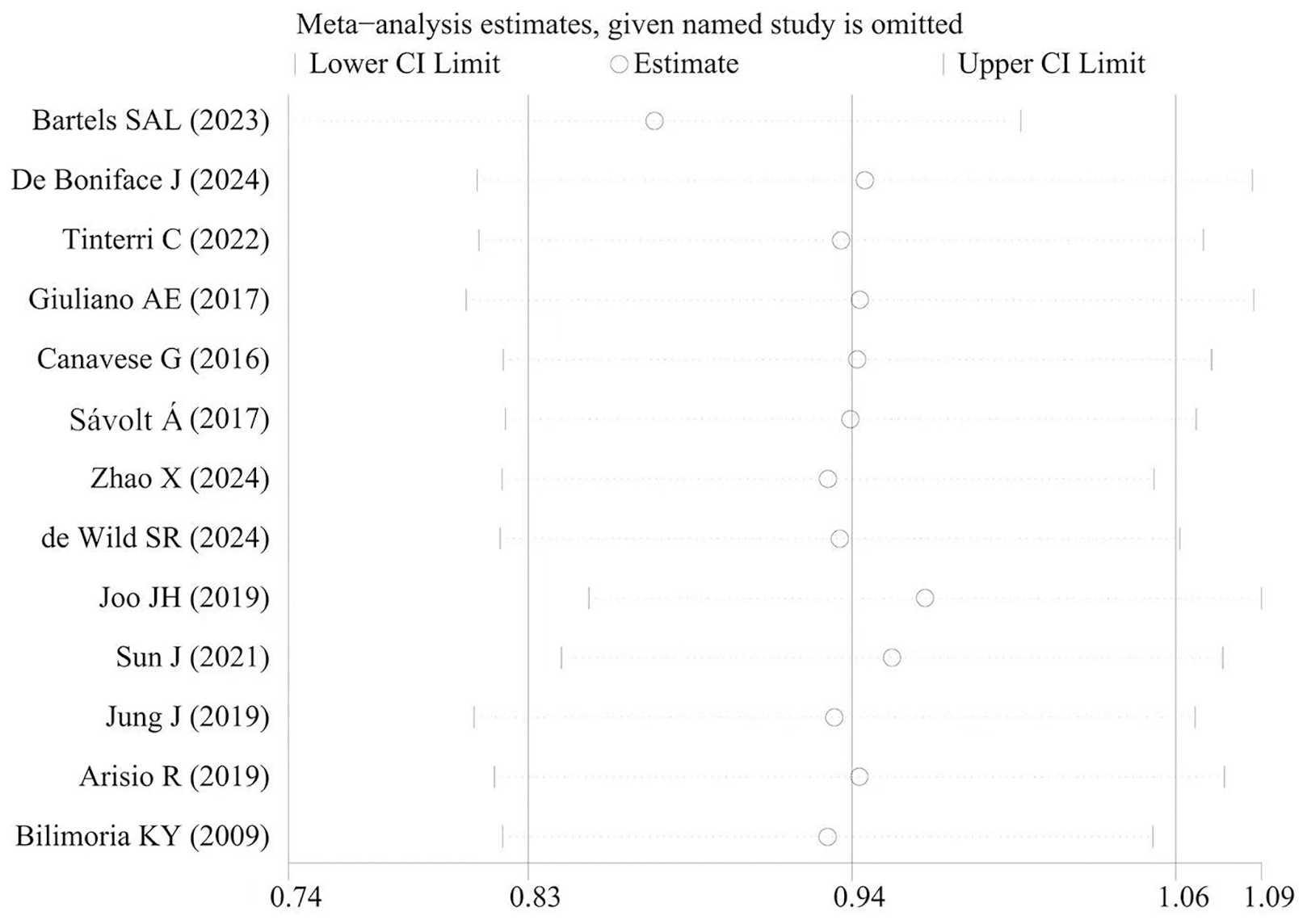

Supplement: Supplementary Figure 3 — Sensitivity analysis of DFS. [file Image3.tif]

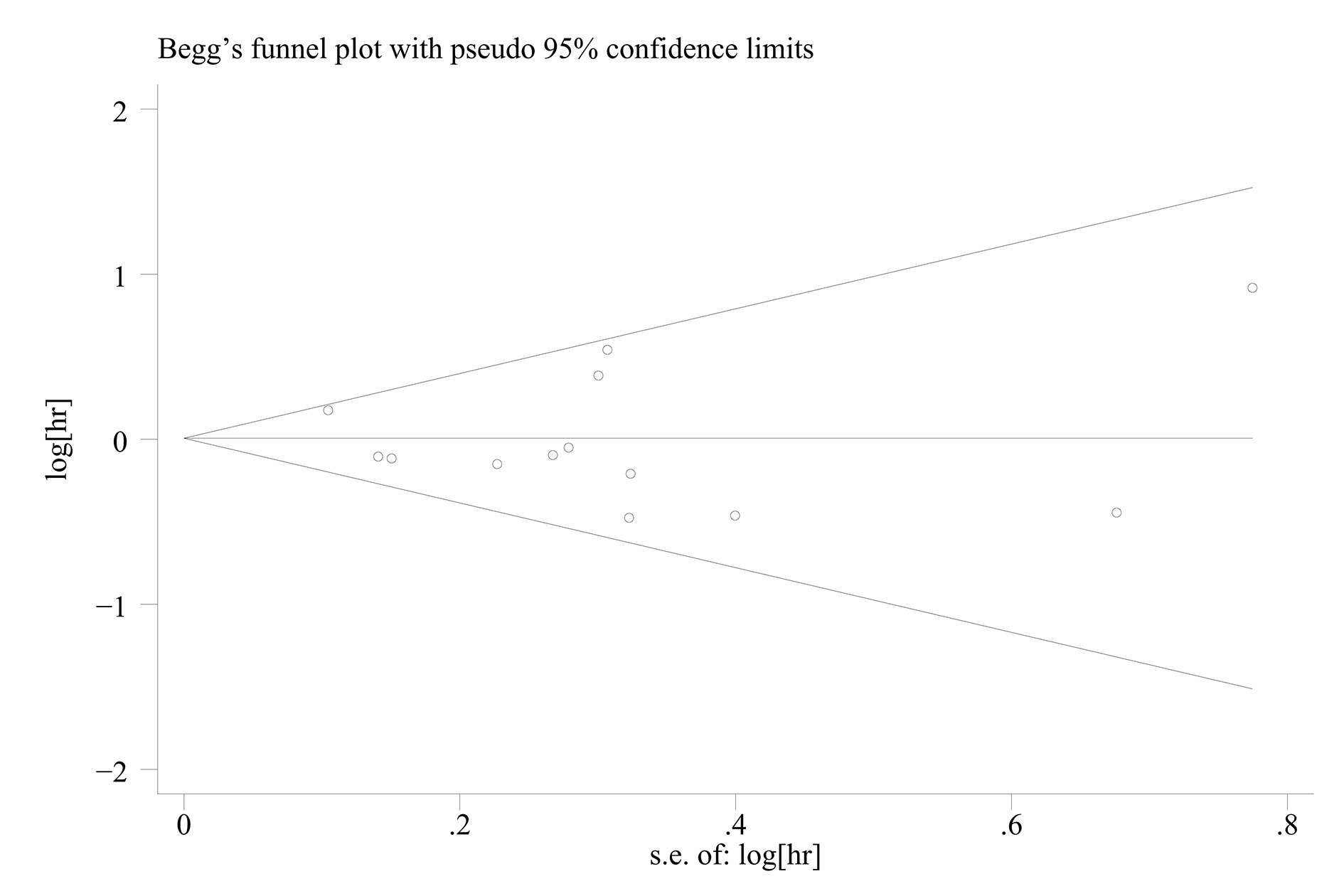

Supplement: Supplementary Figure 4 — Evaluation of publication bias in DFS studies(p=0.951). [file Image4.tif]

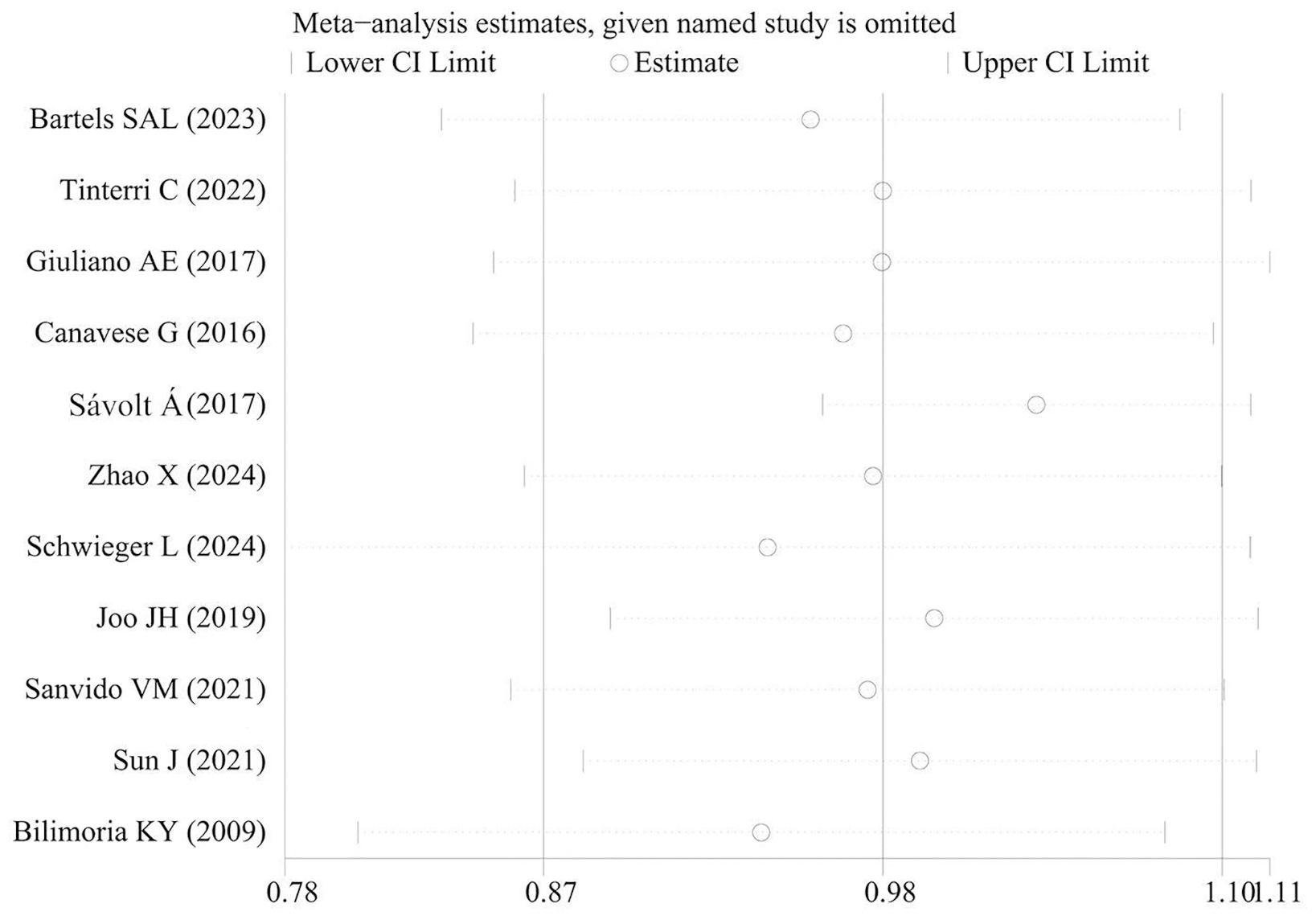

Supplement: Supplementary Figure 5 — Sensitivity analysis of OS. [file Image5.tif]

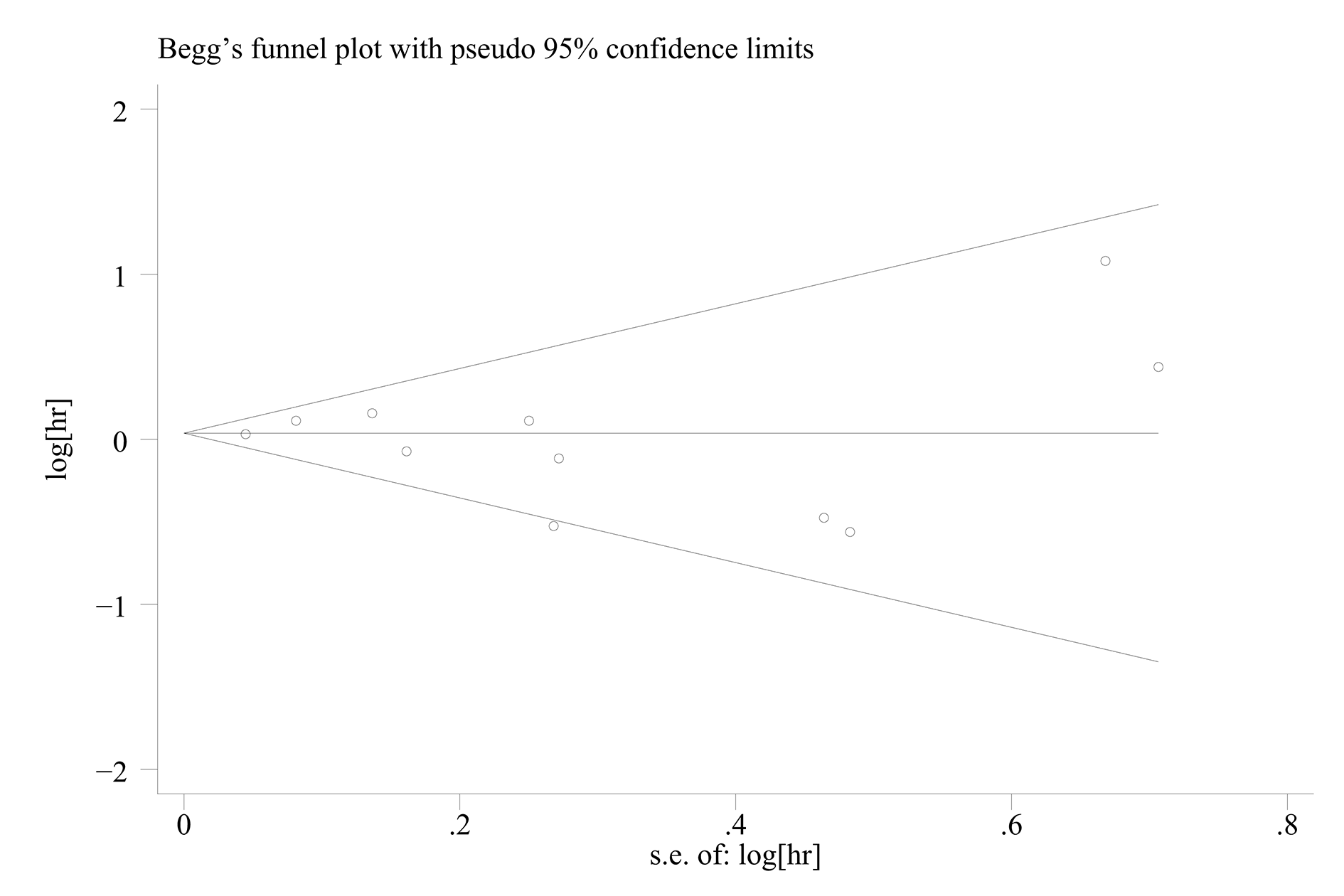

Supplement: Supplementary Figure 6 — Evaluation of publication bias in OS studies(p=0.640). [file Image6.tif]
