# Supplementary material for: Safety analysis of omitting axillary lymph node dissection in early-stage breast cancer with 1–2 sentinel lymph nodes macro-metastases: a meta-analysis
Source: Front Oncol. 2025 Sep 25;15:1620034. doi: 10.3389/fonc.2025.1620034 (PMC12507570; doi:10.3389/fonc.2025.1620034)
Supplement: Supplementary file 7 [file Table1.docx]

**Supplementary Table 1-2. Characteristics of the studies included in this meta-analysis.**

| Author | Year | Median age (year) | | Histological grade | | | | | | | |
| --- | --- | --- | --- | --- | --- | --- | --- | --- | --- | --- | --- |
|  |  |  |  | Experiment arm | | | | Control arm | | | |
|  |  | Experiment arm | Control arm | Grade 1 | Grade 2 | Grade 3 | Missing data | Grade 1 | Grade 2 | Grade 3 | Missing data |
| Bartels SAL | 2023 | 55 | 56 | 154 | 311 | 200 | 16 | 179 | 356 | 192 | 17 |
| De Boniface J | 2024 | 61 | 61 | 243 | 786 | 298 | 8 | 211 | 717 | 263 | 14 |
| Tinterri C | 2022 | 56 | 56 | 49 | 279 | 105 | 0 | 43 | 290 | 104 | 1 |
| Giuliano AE | 2017 | 54 | 56 | 81 | 148 | 87 | 120 | 71 | 158 | 94 | 97 |
| Canavese G | 2016 | 60 | 59 | 5 | 44 | 59 | 2 | 7 | 46 | 60 | 2 |
| Sávolt Á | 2017 | 55 | 55 | 50 | 111 | 69 | NA | 38 | 125 | 81 | NA |
| Zhao X | 2024 | 52 | 52 | 14 | 187 | 33 | NA | 14 | 190 | 30 | NA |
| Schwieger L | 2024 | NA | NA | 1589 | 4360 | 2183 | 295 | 1830 | 5890 | 3435 | 419 |
| De Wild SR | 2024 | 68 | 56 | 55 | 132 | 32 | 2 | 75 | 239 | 111 | 12 |
| Joo JH | 2019 | 49 | 48 | 6 | 105 | 44 | NA | 55 | 823 | 578 | NA |
| Sanvido VM | 2021 | 58 | 58 | 17 | 27 | 12 | NA | 13 | 19 | 9 | NA |
| Sun J | 2021 | 56 | 51 | 18 | 69 | 41 | NA | 20 | 84 | 95 | NA |
| Jung J | 2019 | 49 | 49 | 112 | 401 | 188 | 6 | 111 | 552 | 319 | 8 |
| Arisio R | 2019 | 57 | 54 | 60 | 104 | 47 | NA | 42 | 205 | 159 | NA |
| Bilimoria KY | 2009 | 58 | 56 | 11888 | NA | 6579 | 1750 | 40919 | NA | 30250 | 5928 |

NA, not available.
