# Supplementary material for: Safety analysis of omitting axillary lymph node dissection in early-stage breast cancer with 1–2 sentinel lymph nodes macro-metastases: a meta-analysis
Source: Front Oncol. 2025 Sep 25;15:1620034. doi: 10.3389/fonc.2025.1620034 (PMC12507570; doi:10.3389/fonc.2025.1620034)
Supplement: Supplementary file 8 [file Table2.doc]

**Supplementary Table 2.** Quality assessment of cohort studies included.

| Author, year | **Selection (Out of 4)** | | | | **Comparability**  **(Out of 2)** | **Outcomes (Out of 3)** | | | **Total**  **(Out of 9)** |
| --- | --- | --- | --- | --- | --- | --- | --- | --- | --- |
|  | Representativeness of exposed cohort | Selection of non exposed cohort | Ascertainment  of exposure | Outcome not present at the start of the study |  | Assessment of outcomes | Length of follow-up | Adequacy of follow up of cohorts |  |
| Zhao X, 2024 | 1 | 1 | 1 | 1 | 1 | 1 | 1 | 1 | 8 |
| Schwieger L, 2024 | 1 | 1 | 1 | 1 | 1 | 1 | 1 | 0 | 7 |
| De Wild SR, 2024 | 1 | 1 | 1 | 1 | 1 | 1 | 1 | 0 | 7 |
| Joo JH, 2019 | 1 | 1 | 1 | 1 | 1 | 1 | 1 | 1 | 8 |
| Sanvido VM, 2021 | 1 | 1 | 1 | 1 | 1 | 1 | 1 | 0 | 7 |
| Sun J, 2021 | 1 | 1 | 1 | 1 | 1 | 1 | 1 | 1 | 8 |
| Jung J, 2019 | 1 | 1 | 1 | 1 | 1 | 1 | 1 | 0 | 7 |
| Arisio R, 2019 | 1 | 1 | 1 | 1 | 1 | 1 | 1 | 1 | 8 |
| Bilimoria KY, 2009 | 1 | 1 | 1 | 1 | 2 | 1 | 1 | 1 | 9 |

The cohort studies were assessed by the Newcastle-Ottawa Quality Assessment Scale (NOS) checklist.
