# Supplementary material for: Safety analysis of omitting axillary lymph node dissection in early-stage breast cancer with 1–2 sentinel lymph nodes macro-metastases: a meta-analysis
Source: Front Oncol. 2025 Sep 25;15:1620034. doi: 10.3389/fonc.2025.1620034 (PMC12507570; doi:10.3389/fonc.2025.1620034)
Supplement: Supplementary file 9 [file Table3.docx]

**Supplementary Table 1-1. Characteristics of the studies included in this meta-analysis.**

| Author | Year | Trial name | NCT number | Follow-up (month) | Number of SLNs macro-metastases | | | | | |
| --- | --- | --- | --- | --- | --- | --- | --- | --- | --- | --- |
|  |  |  |  |  | Experiment arm | | | Control arm | | |
|  |  |  |  |  | 1 | ≥2 | Not informed | 1 | ≥2 | Not informed |
| Bartels SAL | 2023 | AMAROS | NCT00014612 | 120 | NA | NA | 419 | NA | NA | 442 |
| De Boniface J | 2024 | SENOMAC | NCT02240472 | 46.8 | 1143 | 192 | NA | 1008 | 197 | NA |
| Tinterri C | 2022 | SINODAR-ONE | NCT05160324 | 34 | NA | NA | 436 | NA | NA | 434 |
| Giuliano AE | 2017 | Z0011 | NCT00003855 | 111.6 | NA | NA | 202 | NA | NA | 228 |
| Canavese G | 2016 | NA | NA | 171.6 | 21 | 7 | NA | 17 | 8 | NA |
| Sávolt Á | 2017 | OTOASOR | NA | 97 | NA | NA | NA | 129 | 54 | NA |
| Zhao X | 2024 | NA | NA | 56.7 | NA | NA | 234 | NA | NA | 234 |
| Schwieger L | 2024 | NA | NA | 74.8 | 6877 | 1550 | NA | 5491 | 6083 | NA |
| De Wild SR | 2024 | NA | NA | 72 | NA | NA | 55 | NA | NA | 392 |
| Joo JH | 2019 | NA | NA | 93 | NA | NA | 123 | NA | NA | 1523 |
| Sanvido VM | 2021 | NA | NA | 42-63.6 | NA | NA | 35 | NA | NA | 40 |
| Sun J | 2021 | NA | NA | 51 | NA | NA | NA | NA | NA | NA |
| Jung J | 2019 | NA | NA | 50 | 520 | 3 | NA | 824 | 124 | NA |
| Arisio R | 2019 | NA | NA | 84.4 | NA | NA | 95 | NA | NA | 322 |
| Bilimoria KY | 2009 | NA | NA | 63 | NA | NA | 16543 | NA | NA | 70512 |

NCT, **national clinical trial;** **NA, not available, SLNs, sentinel lymph nodes.**
